# Diatom-guided bone healing via a hybrid natural scaffold

**DOI:** 10.1016/j.heliyon.2024.e25878

**Published:** 2024-02-11

**Authors:** Mina Mohammadi, Samin Abbaszadeh, Vahideh Nosrati-Siahmazgi, Mahsa Akbari, Saman Rezaei, Kiyan Musaie, Mohammad Reza Eskandari, Hélder A. Santos, Narges Poursina, Mohammad-Ali Shahbazi

**Affiliations:** a Department of Pharmaceutical Biomaterials, School of Pharmacy, Zanjan University of Medical Science, 45139-56184 Zanjan, Iran; b Department of Pharmacology, School of Medicine, Zanjan University of Medical Science, 45139-56111 Zanjan, Iran; c Department of Pharmacology and Toxicology, School of Pharmacy, Urmia University of Medical Sciences, Urmia, Iran; d Department of Pharmaceutical Nanotechnology, School of Pharmacy, Zanjan University of Medical Science, 45139-56184 Zanjan, Iran; e Department of Biomaterials and Biomedical Technology, University Medical Center Groningen, University of Groningen, Antonius Deusinglaan 1, 9713 AV Groningen, the Netherlands; f Department of Pharmacology and Toxicology, School of Pharmacy, Zanjan University of Medical Science, 45139-56184, Zanjan, Iran; g Drug Research Program, Division of Pharmaceutical Chemistry and Technology, Faculty of Pharmacy, University of Helsinki, Helsinki FI-00014, Finland; h Department of Pharmaceutics, School of Pharmacy, Zanjan University of Medical Science, 45139-56184 Zanjan, Iran

**Keywords:** Bone tissue engineering, Three-dimensional scaffolds, Diatom, β-sitosterol, Angiogenesis, Osteoconductive

## Abstract

Bone tissue engineering (BTE) involves the design of three-dimensional (3D) scaffolds that aim to address current challenges of bone defect healing, such as limited donor availability, disease transmission risks, and the necessity for multiple invasive surgeries. Scaffolds can mimic natural bone structure to accelerate the mechanisms involved in the healing process. Herein, a crosslinked combination of biopolymers, including gelatin (GEL), chitosan (CS), and hyaluronic acid (HA), loaded with diatom (Di) and β-sitosterol (BS), is used to produce GCH-Di-S scaffold by freeze-drying method. The GCH scaffold possesses a uniform structure, is biodegradable and biocompatible, and exhibits high porosity and interconnected pores, all required for effective bone repair. The incorporation of Di within the scaffold contributes to the adjustment of porosity and degradation, as well as effectively enhancing the mechanical property and biomineralization. *In vivo* studies have confirmed the safety of the scaffold and its potential to stimulate the creation of new bone tissue. This is achieved by providing an osteoconductive platform for cell attachment, prompting calcification, and augmenting the proliferation of osteoblasts, which further contributes to angiogenesis and anti-inflammatory effects of BS.

## 1 Introduction

Bone tissue engineering (BTE) has garnered significant interest as a novel approach, primarily due to the escalating incidence of bone fractures, which surged by approximately 33.4% from 1990 to 2019 according to global reports [1,2]. The current clinical treatment of bone defects involves the utilization of autografts, allografts, and xenograft implants. However, these approaches are plagued by shortcomings such as limited accessibility, the requirement for multiple invasive surgeries, the potential for disease transmission, sterilization challenges, and inadequate regenerative capability [[3], [4], [5]]. The BTE strategy presents a potential solution to overcome these hurdles. The fundamental objective of BTE is to enhance, sustain, and reinforce the functionality of compromised bones, particularly large bone defects. This is achieved through the deployment of scaffolds that mimic the components, structure, and functions of natural bone. By doing so, these scaffolds facilitate an elevated rate of new bone formation, capitalizing on the innate self-healing attributes of bone tissue [3,6,7]. Scaffolds constitute three-dimensional (3D) structures essential to BTE, encompassing critical characteristics such as biocompatibility, biodegradability, mechanical robustness, bioactivity, osteoinductive potential, and osteoconductive properties. The presence of appropriate porosity, pore dimensions, and interconnected channels within scaffolds create an optimal environment for cellular mobility, the release of growth factors and nutrients, and the expulsion of cellular by-products [[8], [9], [10], [11]].

Several biomaterials have been introduced for the fabrication of scaffolds, including polymers, ceramics, bioglass, metals, and composites. The scaffolds based on biopolymers, such as collagen (Coll) [12,13], gelatin (GEL) [[14], [15], [16], [17]], chitosan (CS) [14,15,18,19], silk [20], alginate (Alg) [15,21], hyaluronic acid (HA) [18,22], and peptides [23] have garnered substantial attention within the BTE realm due to their controllable physicochemical attributes, impressive biocompatibility and biodegradability, facile manufacturing processes, inherent bioactivity, and potential for functionalization to enhance tissue engineering outcomes. In this study, a composite scaffold composed of GEL, CS, and HA biopolymers crosslinked with EDC/NHS via the interaction between carboxylate groups in GEL and HA and amine groups in GEL and CS is prepared. This synergistic assembly is utilized to leverage the strengths of each component to yield favourable physicochemical properties while enhancing scaffold efficacy for BTE. GEL, serving as a central scaffold component, boasts a notable tripeptide sequence RGD (arginine-glycine-aspartic acid), which plays a pivotal role in fostering the attachment, proliferation, and differentiation of osteoblast cells [24]. The incorporation of the RGD sequence within GEL, alongside CS, augments mechanical resilience, biomineralization, and the deposition of calcium by osteoblasts [14,[25], [26], [27]]. Additionally, the inclusion of CS and HA in the range of 0.01–0.1% w/v supports bone regeneration through antimicrobial activity, enhanced cell adhesion, increased water absorption, and favourable osteoconductive and osseointegration behaviours [[28], [29], [30], [31]]. Furthermore, HA stabilizes the receptors of the cell signalling, such as CD44 and RHAMM, that play a role in regulating responses to growth factors and cell migration [32].

Despite the numerous merits of biopolymers, their utilization in BTE is not without challenges, including rapid degradation rates and inadequate mechanical strength. To address these issues, the incorporation of mineral materials is introduced [[33], [34], [35]]. Diatoms (Di) are single-celled algae encased in biomineralized silica frustules, boasting intricate biomimetic hybrid microstructures, extensive multi-scale porosity, a substantial specific surface area, biocompatibility, and negative charge due to abundant free hydroxyl groups in silica [[36], [37], [38], [39], [40], [41]]. These attributes confer impressive biological and mechanical properties [[42], [43], [44], [45], [46]]. Moreover, Di biosilica promotes cell adhesion and proliferation, alkaline phosphatase activity (ALP), and biomineralization, which makes it a promising candidate for BTE [20,[47], [48], [49]].

To further enhance bone healing effects, β-sitosterol (BS), a natural phytosterol structure like cholesterol, imparts angiogenic, antioxidant, anti-inflammatory, antimicrobial, and anti-osteoporosis activities [[50], [51], [52], [53], [54]].

**Fig. 1:**
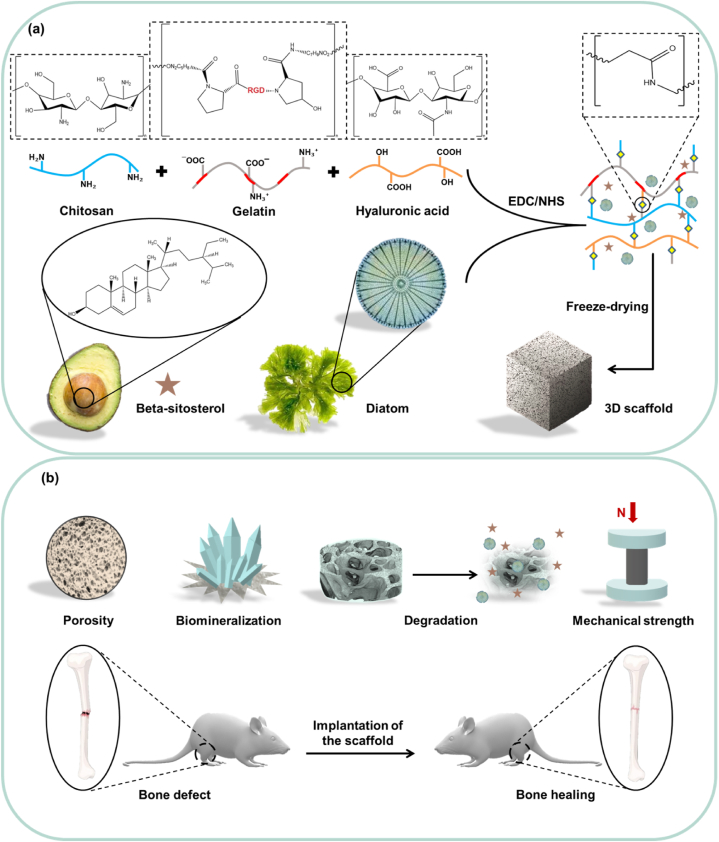
(a) Schematic illustration of the preparation procedure of GCH-Di-S hybrid scaffold. (b) The advantages of the prepared scaffold, including desirable porosity, biomineralization, degradation, facile drug incorporation, and high mechanical strength lead to the enhanced bone regeneration.

Although the separate functions of Di and biopolymer are reported for BTE, there is no report on the mixture of these biopolymers together and blended with Di for bone regeneration. Moreover, the scaffold enhances the biological function by promoting osteogenesis. The present study aims to create a biosafe 3D GCH-Di-S scaffold to foster bone regeneration through its commendable biodegradability, mechanical strength, suitable porosity, biomineralization capabilities, and propensity for osteogenesis (Fig. 1a–b).

## 2 Method & materials

### 2.1 Preparation and characterization of GCH-Di hybrid scaffold

The freeze-drying method was used to prepare the GCH scaffold [29,55]. First, 7 ml of GEL (Merck, Germany) (5% wt.v^−1^ in deionized water (DW)) was combined with 1 ml of HA (sodium salt, high molecular weight, Bloomage Freda Biopharma, China) with the concentration of 0.05% wt.v^−1^ in DW, followed by stirring at 1000 rpm for 5 min to obtain a homogeneous solution. Then 2 ml of CS (high molecular weight, Sigma-Aldrich, Germany) with the concentration of 3% wt.v^−1^ in 1% v.v^−1^ acetic acid (Merck, Germany) was added to the above solution at 50 °C, followed by stirring at 1500 rpm at the above temperature for 20 min. Finally, the mixture was transferred to a 15-mm diameter cylindrical mold and crosslinked by adding 0.5 ml of 660 mM 1-ethyl-(3,3-dimethyl aminopropyl)-carbodiimide (EDC; prepared in 50 mM MES buffer) and 0.5 ml of 176 mM N-hydroxy succinimide (NHS; prepared in 50 mM MES buffer). The sample was immediately mixed and vacuumed for 2 min to remove bubbles before freezing at −80 °C for 12 h (NEW BRUNSWICK SCIENTIFIC, Ultra low temperature freezer, USA). The scaffold was freeze-dried (EYELA FDU-2100, Japan) at −80 °C for 48 h. Di was then added to the GCH solution in 2, 5, and 10% wt.wt^−1^ ratio based on the total content of polymers; afterward, the mixture of the composite was transferred to the mold followed by an above-explained procedure to obtain the scaffold. Based on the results of characterizing GCH-Di scaffolds, including measurements of porosity, swelling, degradation, and mechanical strength, the scaffold with GCH-Di 10% was used for loading BS (Sigma-Aldrich, Germany), dissolved BS at 0.1% wt.wt^−1^ (ratio based on the total content of scaffold) in a minimum amount of absolute ethanol (Merck, Germany) was added to the above mixture composite and mixed to ensure uniform distribution. The ethanol was evaporated by leaving the sample at 50 °C in the water bath for 2 min. Similar to the above scaffolds, the mixture was transferred to the mold, and then the GCH-Di-S scaffold was obtained following the previously described method.

The gelling time of GCH-Di-S was determined by adding a 200 μl of EDC/NHS solution in a tube containing about 1 ml of GCH-Di-S hydrogel scaffold at temperature room and the time needed for gel formation was found by visual observations. The morphology of scaffolds (GCH, GCH-Di with 2, 5, and 10% Di, as well as GCH-Di-S) and pure Di were examined by scanning electron microscopy (SEM; FEI Quanta 200, USA) and energy dispersive X-ray (EDX) was used for elemental analysis. The porosity of the samples was determined by the liquid displacement method [56]. All prepared scaffolds (GCH, GCH-Di with 2, 5, 10% Di, and GCH-Di-S) were prepared and freeze-dried, then were first immersed in 10 ml of absolute ethanol then the scaffolds were removed from ethanol after 20 min and 24 h. The porosity ratio was evaluated by using Eq. (1):

(1) $$\text{Porosity}\%=\frac{\left(V_{1}-V_{3}\right)}{\left(V_{2}-V_{3}\right)}\times 100$$

where, V_1_ is the volume of ethanol used to immerse the scaffold, V_2_ is the volume of the ethanol with the saturated scaffold, and V_3_ is the remaining ethanol volume when the scaffold is removed. The interaction between GEL, CS, HA, and Di was investigated by Fourier-transform infrared spectroscopy (FT-IR) (BRUKER TENSOR 27, Germany) and was recorded in the wavelength range of 4000–500 cm^−1^ at room temperature. X-ray diffraction spectrometry (XRD; Philips PW1730, Netherlands) was used to study the crystallinity of scaffolds at room temperature with the scan range of 2θ = 10°–80°.

### 2.2 Thermal analysis

Thermal Gravimetric Analysis (TGA; TA Q 600 USA) and derivative thermogravimetry (DTG) analysis were conducted to study the thermal stability and phase transitions with the heating temperature range 40–850 °C and a temperature ramp of 10 °C.min^−1^ under nitrogen atmosphere.

### 2.3 Swelling and degradation

To calculate the percentage of swelling ratio, scaffolds (GCH, GCH-Di with 2, 5, 10% Di, and GCH-Di-S) were prepared and freeze-dried, then were immersed in 20 ml of phosphate buffer saline (PBS; pH 7.4) followed by incubation at 37 °C for 24 h. At different time points (0, 1, 2, 4, 8, and 12 h), samples were removed from buffer and placed on the filter paper to eliminate the excess water from the surface. The wet weight was then measured. Swelling ratios were calculated using Eq. (2):

(2) $$\text{Swelling}\%=\frac{\left(W_{1}-W_{0}\right)}{\left(W_{0}\right)}\times 100$$

where, W_1_ is the initial weight of the scaffold freeze-dried and W_2_ is the weight of the scaffold after being removed from the buffer.

To measure the degradation ratio, the scaffolds (GCH, GCH-Di with 2, 5, 10% Di, and GCH-Di-S) were prepared and freeze-dried, then were immersed in 20 ml PBS (pH 7.4) and incubated at 37 °C for 14 days. At certain times (0, 1, 2, 4, 8, and 12 h, along with 1, 2, 3, 4, 5, 6, 8, 10, 12, and 14 d), all the scaffolds were removed and dried in the oven at 50 °C for 10 h. Degradation ratios were calculated using Eq. (3):

(3) $$\text{Degradation}\%=\frac{\left(W_{0}-W_{1}\right)}{\left(W_{0}\right)}\times 100$$

where, W_0_ is the primary weight of the freeze-dried scaffold and W_1_ is the weight of the scaffold after being removed from the buffer and dried.

### 2.4 Mechanical properties

To evaluate the mechanical properties, scaffolds (GCH, GCH-Di with 2, 5, 10% Di, and GCH-Di-S) were cut into 15 mm in diameter and 22 mm in length, and subjected to the vertical force in the cross-section of the cylinders with a speed of 10 mm min^−1^ and a maximum load of 3000 N until failure at room temperature (SANTAM DBBP-500, Korea). The stress-strain curves of all the samples were drawn, and by calculating the slope of the linear part of the graphs, the mechanical properties and Young's modulus of the scaffold were obtained.

### 2.5 Hemocompatibility

The hemocompatibility test was performed for Di and scaffolds (GCH, GCH-Di 10%, and GCH-Di-S). The samples were first sterilized by ultraviolet irradiation for 20 min. Within 2 h after fresh blood sampling inside the heparin tube, red blood cells (RBCs) were isolated by mixing 5 ml of the blood with 10 ml of PBS; pH 7.4, and centrifuging at 2500 rpm (6 min) for five times. Then, 5% hematocrit (HCT) suspension was prepared by mixing 2 ml of the RBC pellet with 38 ml of PBS; pH 7.4. The hemolytic effect of the composites was evaluated by adding a ratio of 1:4 of the 5% hematocrit and the different concentrations of composite particles (100, 200, 500, and 1000 μg ml^−1^) and Di concentrations (200, 300, 400, and 500 μg ml^−1^) in PBS; pH7.4. Next, all samples were incubated at room temperature for 2, 4, 8, and 24 h. Then, the samples were centrifuged at 6000 rpm for 5 min, and 150 μl of the supernatant was transferred to 96-well plates (SPL 96 Flat Bottom Transparent Polystyrene, Korea) and read at 540 nm (TECAN infinite M200 microplate Reader, Switzerland) to quantify the lysed hemoglobin (HGB). The percentage of hemolysis was calculated using Eq. (4):

(4) $$\text{Hemolysis}\%=\frac{\left(A_{s}-A_{n}\right)}{\left(A_{p}-A_{n}\right)}\times 100$$

where, A_s_ is the absorbance of the sample, A_n_ is the absorbance of the negative control (PBS; pH 7.4), and A_p_ is the absorbance of the positive control (DW).

### 2.6 Biomineralization assay

The biomineralization of the GCH-Di-S scaffold was evaluated by immersing the samples in 10 ml simulated body fluid (SBF) solution according to the ISO 23317 (Implants for surgery-*in vitro* evaluation for the apatite-forming ability of implant materials) [57]. The scaffolds were cut into 15 mm in diameter and 2 mm in length and immersed in SBF at 37 °C for 14 and 28 days. Next, the samples were freeze-dried at −80 °C for 36 h. The surface morphology of the scaffold was determined by SEM, and the Ca:P ratios were evaluated using an EDX.

### *2.7* *In vivo* toxicity and bone formation studies

To evaluate the effects of scaffolds on bone regeneration, healthy adult Wistar male rats weighing 250–300 g were divided into five groups (N = 3 per each time point of study), including a) control group without any bone defect, b) bone defect in the tibia without scaffold implantation (surgery group), c) bone defect in the tibia and GCH implantation, d) bone defect in the tibia and GCH-Di 10% implantation, and e) bone defect in the tibia and GCH-Di-S implantation.

Initially, all rats were anesthetized by intraperitoneal (IP) injection of 0.25 ml of ketamine (50 mg ml^−1^) and xylazine (20 mg ml^−1^) cocktail 6:4 v.v^−1^. After shaving and slitting the hair of the left leg in animals and creating a surgical cut on the skin, round hole defects were created using a bone drill (Universal 500 W, Turkey) with Wilson nail drill 5,000,209 at the tibia exactly below the knee. The scaffolds were sterilized under UV irradiation for 20 min, and inserted at the defect site by the press-fit method, followed by suturing the skin. The rats were sacrificed 3, 5, and 7 weeks after implantation and tibia bone of all groups was removed and fixed in 4% neutral-buffered formalin (Sigma-Aldrich, Germany) for micro-computed tomography (μ-CT) and histological examinations. The μ-CT analysis was performed using a μ-CT scanner (CBCT Giano 3D, New Tom, Italy) under a source voltage of 110 kV, a current of 9.01 mA, and an exposure time of 3.5 s 3D remodelling was conducted using the NNT software. For histology analysis, the bone slides were stained using hematoxylin and eosin (H&E) and Masson's Trichrome (MT) under optical microscopy (Olympus BX61, Japan) after μ-CT.

For the *in vivo* toxicity test, animal blood was collected 5 weeks after scaffold implantation for analyzing different hematological and biochemical parameters. To do histopathological analysis, the main organs (liver, spleen, and kidney) were removed, washed with PBS (pH 7.4) for 2 min, and fixed in 4% buffered formalin, followed by H&E staining.

During the *in vivo* studies, all the rats were housed in standard steel cages in a temperature-controlled animal room at 22 ± 2 °C with 50 ± 10% humidity and a 12-h light/dark cycle**.** The animal study was confirmed by the Ethics Committee of Zanjan University of Medical Sciences (IR.ZUMS.REC.1400.301).

### 2.8 Statistical analyzes

All data were reported as mean± standard deviations (SD). One way ANOVA test and Tukey post-hoc test were used for the statistical analysis of data, as well as *p < 0.05, **p < 0.01, and ***p < 0.001 were used to determine differences between groups.

## 3 Results and discussion

### 3.1 Physicochemical characterization of scaffolds: gelation time, morphological properties, porosity, EDX, FT-IR, and XRD studies

**Fig. 2:**
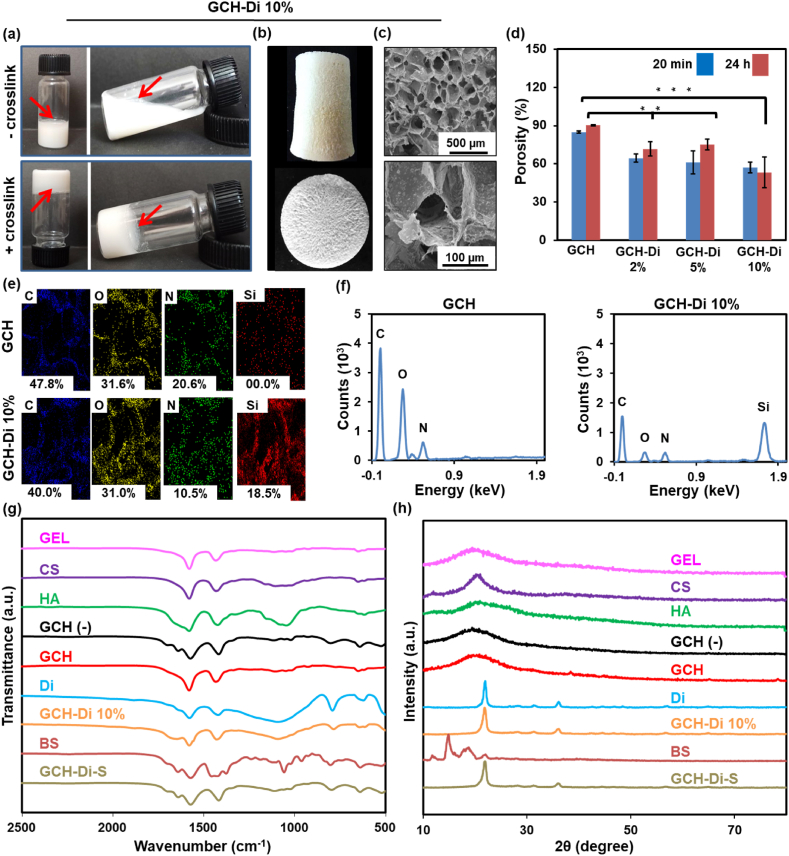
Characterization of scaffolds; The GCH (−) represents the scaffold non-crosslinked, the GCH represents the final scaffold with crosslinked, the GCH-Di 2% represents the GCH scaffold with incorporate 2% Di and crosslinked, the GCH-Di 5% represents the GCH scaffold with incorporate 5% Di and crosslinked, the GCH-Di 10% represents the GCH scaffold with incorporated 10% Di and crosslinked, and the GCH-Di-S represents the GCH scaffold with incorporated 10% Di, BS, and crosslinked. (a) Photographs of GCH-Di 10% hydrogel in the presence and absence of EDC/NHS as the crosslinker. (b,c) Macroscopic images of GCH-Di 10% scaffold in different viewpoints and corresponding SEM images with different magnifications. (d) Porosity analysis of the prepared scaffolds including GCH, GCH-Di with 2, 5, and 10% of Di. Statistical analysis calculated by one way ANOVA with the level of significance set at * p < 0.05, **p < 0.01, **p < 0.001. (e,f) Elemental mapping and EDX analysis of the GCH and GCH-Di 10% scaffolds. (g–h) FT-IR and XRD of the pure materials (GEL, CS, HA, Di, and BS), GCH (−), GCH, GCH-Di 10%, and GCH-Di-S scaffolds. Data are represented as the mean ± SD (N = 3).

The gelling systems are fluid formulations that form semi-solid contracts after adding a crosslinker into the hydrogel; for this purpose, the gell formation of hydrogel scaffolds was studied with the tube inversion method (Figs. 2a and S1) [58]. The gel formation of the GCH hydrogel with and without EDC/NHS as the crosslinker was assessed by using the tube inversion method. The addition of the crosslinker to the solution resulted in hydrogel formation of approximately 10 s without any flow in the bottom-up position through the formation of amide bonds between carboxyl/amine groups of GEL, CS, and HA. However, the solution with no crosslinker showed a free flowing at the same condition. Noteworthy, the addition of Di and BS to the solution showed no effect on hydrogel formation. Macroscopic imaging of open-porous GCH-Di with 0, 2, 5, and 10% Di and GCH-Di-S scaffolds were successfully conducted after freeze-drying (Figs. 2b and S2). The SEM images of Di and the prepared scaffolds (GCH and GCH-Di with 2, 5, and 10% Di) are shown in Figs. S3 and 2c. The SEM image of the pure Di revealed a disc-shape structure with a length of 20–40 μm, in which the increment of Di amount in the smooth surface of GCH resulted in the increased turbidity of polymer solution and roughness of the GCH-Di 10% scaffold surface as well as reduction of the porosity from 90.0 ± 0.3% to 53.0 ± 1.2% due to the space occupied by Di within the structure (Fig. 2d). Furthermore, the GCH-Di 10% scaffold revealed the average pore sizes of about 240 μm, obtained from SEM images, in the desirable range of 100–600 μm pore size needed for mineralized bone growth [59]. The porous interconnected network of the scaffold mainly accelerates the penetration of oxygen and cell nutrients and removes metabolites in order to regenerate tissue.

Figs. 2e and f, and S4 indicate the elemental mapping and EDX analysis of the GCH and GCH-Di 10% scaffolds. The GCH scaffold was comprised of 47.8 wt% of C, 31.6 wt% of O, and 20.6 wt% of N. In the structure of the GCH-Di 10% scaffold, these amounts changed to 40.0%, 31.0%, and 10.5% for C, O, and N. Also, Si (18.5%) was detected, confirming the increased amount of Si by loading Di into the scaffold (Table S1).

FT-IR was used to determine the chemical composition and confirm the crosslinking of polymers in the GCH-Di 10% hybrid by EDC/NHS chemistry (Fig. 2g and Table S2). In the spectrum of GEL, the bands at 1583 cm^−1^ and 1440 cm^−1^ represent the amide groups and C]O stretching vibration carboxylate groups, attributed to the amino acids backbone. The band at 1571 cm^−1^ of CS corresponded to the N–H stretching amine groups. The broad bands of HA observed at 1000-1200 cm^−1^ and 1600 cm^−1^ were related to the C–O groups of alcohol I & II and C–O stretching carboxylate groups. In the GCH scaffold, percentage of GEL, CS, and HA were ⁓85.3% wt.wt^−1^, ⁓14.6% wt.wt^−1^, and ⁓0.1% wt.wt^−1^, respectively, meaning the final spectra sample could be more affected by the presence of GEL. The spectra comparison between non-crosslinked and crosslinked GCH scaffolds are shown in Fig. S5, demonstrating that the amide I (1573 → 1581 cm^−1^) and amide II (1417 → 1435 cm^−1^) bands were slightly shifted due to crosslinking reaction between carboxylate groups of GEL and HA with amine groups of GEL or CS via EDC/NHS [15,60,61].

The percentages of GEL, CS, HA, and Di in the final freeze-dried GCH-Di 10% scaffold were 24.8%, 4.3%, <0.1%, and 70.9%, respectively. A strong and broad (1090 cm^−1^) and a sharp (1640 cm^−1^) band were observed due to the high percentage of Di in the GCH-Di 10% scaffold, which is attributed to the asymmetric stretching vibration of Si–*O*–Si bonds and bending vibration of O–H in both the spectra of the pure Di and the GCH-Di 10% scaffold, confirming the physically loaded Di into the scaffold [62]. Strong and sharp bands of BS were observed at 1631 cm^−1^, 1580 cm^−1^, and 1460 cm^−1^, corresponding to the Stretching vibration of C]C, Bending vibration of O–H, and Bending vibration of isopropyl, owing to the slight amount of BS in the GCH-Di-S scaffold, no effect on the spectrum was observed [63].

XRD analysis is used to specify the structure of materials, such as crystalline or semi-crystalline structure. XRD spectra of pure initial material and scaffolds are presented in Fig. 2h and Table S3. XRD spectra evaluations indicated the amorphous structure of pure GEL and HA broad peaks in 20.1° and 20° as well as semi-crystalinity of CS with major broad peaks at 10.5° and 20.2°, due to the contribution of intermolecular hydrogen bonding with free –NH_2_ groups inside the molecular structure [64]. The XRD diffraction peak of the GCH scaffold demonstrated amorphous structure due to the presence of GEL as the main component, and crosslinking had no effect on the crystallinity of the scaffold. The XRD pattern of the pure Di revealed its crystalline structure with major diffraction peaks at 21.8° and 36.1° [42,62,65], which is related to the crystalline form of silica in Di. The characteristic peaks of Di were observed in the XRD spectra of the GCH-Di 10% scaffold (JCPDS card number 96-900-8230). The pure BS shown crystalline structure with multi peaks at 10°,15°, and 18.3°, due to slightly percentage of the BS in the GCH-Di-S scaffold (JCPDS card number 96-900-8227) was no affected in the pattern.

### 3.2 Thermal analysis

**Fig. 3:**
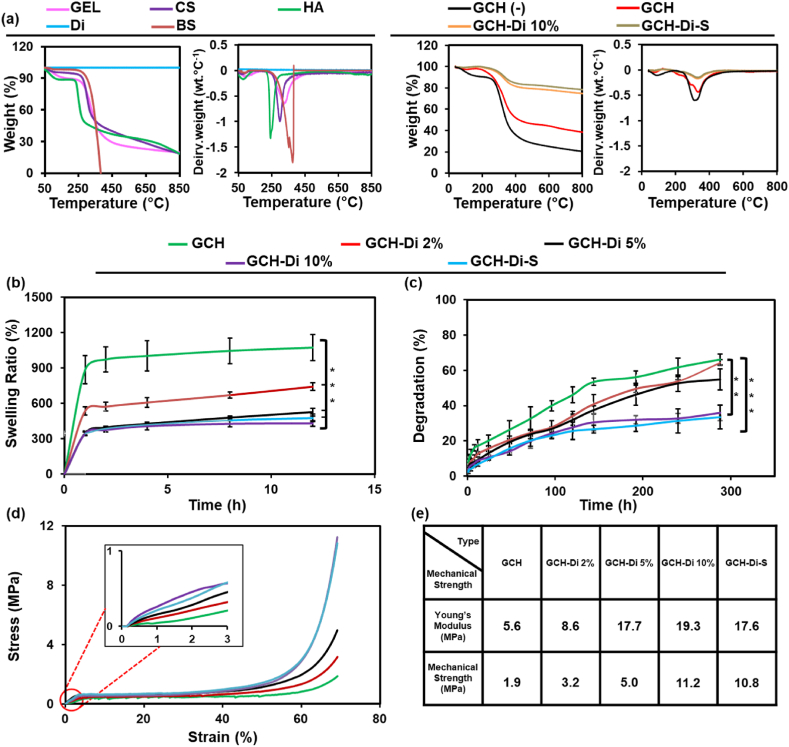
(a) TGA and DTG of the pure materials (GEL, CS, HA, Di, and BS), GCH (−), GCH, GCH-Di 10%, and GCH-Di-S scaffolds. (b–e) Swelling, degradation, and stress-strain curves of the GCH, GCH-Di with 2, 5, and 10% of Di, and GCH-Di-S scaffolds. Data are represented as the mean ± SD (N = 3). Statistical analysis performed by one way ANOVA with the level of significance set at * p < 0.05, **p < 0.01, ***p < 0.001.

TGA curves of GEL, CS, HA, Di, BS, GCH no crosslinker, GCH, GCH-Di 10%, and GCH-Di-S are shown in Figs. 3a and S6 and S7. Two distinct stages of weight loss during heating for all pure materials (except the Di) and GCH with and without crosslinker were observed. In the first stage, weight loss of about 2–11% was observed at the temperature of 150 °C due to the loss of weakly hydrogen-bonded water molecules (dehydration process) [42]. The water content of HA is higher than GEL and CS owing to the presence of numerous hydrophilic hydroxyl and carboxyl groups of the HA [60]. T10% (temperature for 10% weight loss) of HA was appeared at 108 °C. In the second stage for the aforementioned samples, the weight loss occurred at the temperature of 200–350 °C corresponding to the degradation of polar groups such as carboxyl, amine, and amide groups in biopolymers. TGA results show that the Di frustule did not experience any weight change due to the high thermal stability of pure silica content [42,62,66]. The main weight loss of BS started at 280 °C and was totally decomposed at 400 °C with no residue and converted to oxidized products, owing to unstable structure at high temperature [51]. TGA analysis of the GCH scaffold had nearly 62.8% compared GCH no crosslinker scaffold 80.5% weight loss at 850 °C, which demonstrated that chemical crosslinking reaction increased the thermal stability and these losses of weight were probably related to the polymeric backbone [67,68]. The decomposition of the GCH-Di 10% and GCH-Di-S scaffold were about 25.6% and 22.5%, respectively, express their significant thermal stability at 50–850 °C, due to the high amount of Di in the scaffolds, which started at 220 °C and ended at 615 °C as well as T10% their appeared about at 330 °C.

Meanwhile, DTG analysis of the GCH scaffold demonstrated that the maximum degradation rate was about −0.42% °C^−1^ at 338 °C in comparison to GCH no crosslinker scaffold −0.60% °C^−1^ at 310 °C, confirming the improved thermal stability of the chemical crosslinking reaction. Moreover, the GCH-Di 10% scaffold showed reduced rate of the maximum degradation (−0.15% °C^−1^) at 340 °C, certified development of thermal stability by addition of Di (Fig. S7).

### 3.3 Swelling

The hydrophilicity of the GCH, GCH-Di with 2, 5, 10% Di, and GCH-Di-S scaffolds significantly modifies cell adhesion, proliferation, and rehydration capacity; thus, it appears necessary for the efficiency of scaffolds for application in tissue engineering including BTE [60,69,70]. Therefore, calculating and studying the swelling ratio in PBS would predict the interaction between the prepared scaffolds with the surrounding body fluid [71]. The swelling percentage of scaffolds was evaluated over 12 h of incubation at 37 °C by immersion method, as shown in Fig. 3b. The results indicated the highest amount of water absorption for GCH in all time points (Fig. S8), owing to the presence of several polar groups, such as hydroxyl, carboxyl, and amine in the GEL, CS, and HA molecules, which render the scaffold high hydrophilicity, resulting in increased water absorption [29,60]. However, the water capacity of GCH-Di 10 % was decreased to 300% with increasing the percentage of Di, while it was still in the desirable range for BTE application. Indeed, loading of the Di resulted in occupying the free spaces of the scaffold network and reduced the water absorption capacity.

### *3.4* *In vitro* degradation

Degradation behavior of the GCH, GCH-Di with 2, 5, 10% Di, and GCH-Di-S scaffolds play an essential role in bone regeneration, where matrix degradation can support cell attachment, growth, and tissue regeneration without releasing harmful metabolic products. The biodegradation rate of the scaffolds depends on the biomaterial's nature and the processing used to manufacture 3D scaffolds [[71], [72], [73], [74]]. All the GCH, GCH-Di with 2, 5, 10% Di, and GCH-Di-S scaffolds displayed a significant loss of mass during the immersion period (Fig. 3c). The GCH scaffold degraded after five days due to the hydrolysis of numerous functional groups of GEL and CS polymers as the main compounds of the GCH scaffold, while the GCH-Di 10% and GCH-Di-S scaffolds degraded after 10 days [27,60]. The presence of Di prevents water from rapidly penetrating into the scaffold, and the degradation rate decreases by increasing the Di content in the scaffold [75]. Moreover, the degradation rate of scaffolds increased over time during the 14 days of study.

### 3.5 Mechanical property

The high mechanical strength provides mechanical-cell support at the expected level between the matrix and the tissue and prevents the scaffold break during bone regeneration [76,77]. As shown in Fig. 3d, the stress-strain curves revealed the deformation behavior of scaffolds, first-stage linear elastic behavior at low strain, second-stage sequential pore collapse correlated stress reduction at intermediate strains, and third-stage a plastic behavior plateau with following strain stiffening at high strain. We obtain a compressive strength of 1.9 MPa, and Young's modulus of 5.6 MPa for the GCH scaffold. Moreover, the effect of Di addition into the GCH scaffolds with different amounts of 2, 5, and 10% Di was assessed. The GCH-Di 10% scaffold exhibited the highest compressive strength (11.2 MPa) and Young's modulus (19.3 MPa) due to the stiffness character of Di (Fig. 3e) [20,48].

These results followed porosity and degradation data, which showed that adding Di decreased degradation rate and porosity to an appropriate range, while increased mechanical strength led to a better scaffold for bone regeneration [75,78]. Therefore, based on the results of porosity ratio, swelling ratio, degradation ratio, and mechanical properties of GCH, GCH-Di with 2, 5, 10% Di scaffolds, the GCH-Di 10% scaffold was chosen to load BS and perform hemocompatibility and *in vivo* study.

No significant reduction in compressive strength and Young's modulus was observed for GCH-Di-S scaffold, indicating that BS addition had no effect on the strength of the scaffold. On the other hand, many bones such as the skull and hands are non-bear loading, and fabrication of the obtained scaffolds is suitable for application in these bones.

### 3.6 Hemocompatibility

**Fig. 4:**
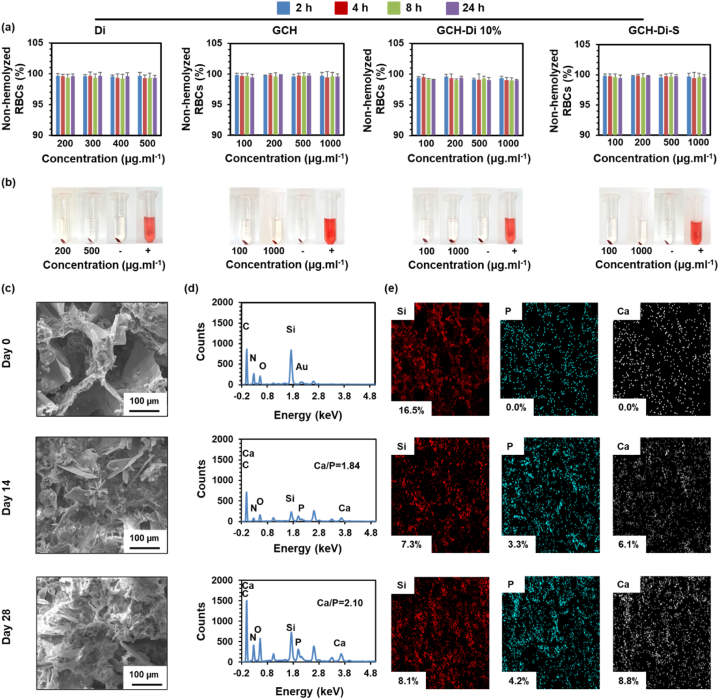
(a–b) Non-hemolyzed RBCs percentage of Di, GCH, GCH-Di 10%, and GCH-Di-S, and corresponding photographs. Data are represented as the mean ± SD (N = 3). Statistical analysis performed by one way ANOVA with the level of significance set at * p < 0.05, **p < 0.01, ***p < 0.001. (c–e) SEM images, EDX and elemental mapping analysis for biomineralization of the GCH-Di-S scaffolds on days 0, 14, and 28 incubated in the SBF. Data are represented as the mean ± SD (N = 3).

Hemocompatible materials with no adverse interaction on lysis or coagulation of red blood cells are of interest properties for clinical applications [79]. To evaluate the hemocompatibility of pure Di and scaffolds (GCH, GCH-Di 10%, and GCH-Di-S), the hemolysis ratio of RBCs was determined (Fig. 4a). The quantitative results of all the samples displayed excellent hemocompatibility with the hemolytic index between 0.11 and 1.02%, which is less than the allowed level for biomaterials (2%) according to ASTMF756-00 (2000) standard [80,81]. The corresponding hemolysis photographic images of Di and scaffolds are displayed in Fig. 4b. All samples were the transparency of the supernatant like the negative control group (PBS) and the red colour of the positive control (DW) owing to the complete lysis of RBCs.

### 3.7 Biomineralization

The biomineralization activity of the GCH-Di-S scaffold was studied *in vitro* for up to 28 days in SBF, as shown in Fig. 4c–e. After incubation of the GCH-Di-S scaffold in SBF, most of the pores were coated by scale-like crystals of hydroxyapatite [82]**.** The SEM results indicated that the presence of Di in the scaffold caused the new bone formation and mineralization at the primary step due to the facilitated accumulation of CaP on the surface of the scaffold [69]. Indeed, the ratio of Ca/P is 1.67, proving the successful coating of hydroxyapatite (stoichiometric Ca_5_(PO4)_3_(OH)) on the scaffold but under conditions such as CO_2_ percentage and temperature, non-stoichiometric hydroxyapatite is produced, the EDX results of the GCH-Di-S scaffold exhibited Ca/P ratio from 1.84 to 2.10 on days 14 and 28, due to the substitution of some PO_4_^−3^ ions by CO_3_^−2^ ions [82]. As shown in Fig. 4d, Ca, P, and Si have clear peaks in the EDX spectrum of the GCH-Di-S scaffold, which revealed the time-dependent increment of the Ca/P ratio and reduction of Si percentage, indicating biomineralization of CaP crystals. A summary of the EDX data is presented in Table S4.

Two mechanisms of hydroxyapatite crystals form on the surface of the GCH-Di-S scaffold were described. The first suggested mechanism is the electrostatic interaction between Ca^+2^ ions and Si–OH groups of Di that provides a positive charge on the surface of the scaffold, which further attracts PO_4_^−3^ ions to form CaP [16,69]. The second mechanism is attributed to the electrostatic attraction between carboxyl groups of polymeric scaffold and the Ca^+2^ ions present in SBF. Meanwhile, amino groups of the polymeric scaffold with positive charge accelerate the combination of PO_4_^−3^ ions of SBF, resulting in the nucleation of hydroxyapatite crystals [83], suggesting the polymer constitution of scaffold along with Di possess the excellent potential for bone regeneration [43,47,84].

### *3.8* *In vivo* toxicity

**Fig. 5:**
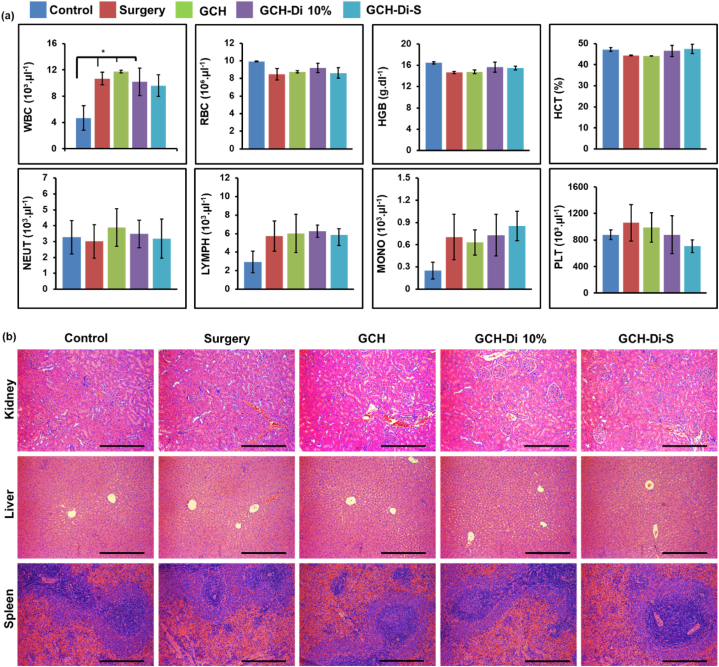
*In vivo* toxicity analysis. (a) Hematological analysis of the scaffolds (GCH, GCH-Di 10%, and GCH-Di-S) compared with control and surgery groups, 5 weeks after the implantation in the defect site of the tibia bone. Data are represented as mean ± SD (N = 3). Statistical analysis calculated by one way ANOVA with the level of significance set at * p < 0.05, **p < 0.01, ***p < 0.001. (b) Histological analysis of organs (kidney, liver, and spleen) of the above-mentioned groups stained with H&E. Scale bar = 100 μm.

The *in vivo* toxicity of the GCH, GCH-Di 10%, and GCH-Di-S scaffolds were evaluated by placing the implants in the defect site of the tibia bone after 5 weeks. As shown in Figs. 5a and S9, no significant difference was observed for hematological factors, including red blood cells (RBCs), hematocrit (HCT), hemoglobin (HGB), and platelet (PLT), except the number of white blood cells (WBCs), which increased significantly for all group compared to the control group, due to immune response and inflammation caused by surgery. Moreover, no significant difference was observed for biochemical parameters, including blood urea nitrogen (BUN), creatinine (CREA), lactate dehydrogenase (LDH), alkaline phosphatase (ALP), total protein (TP), albumin (ALB), calcium (Ca), and phosphor (Ph). Furthermore, the histological analysis of the organs of rats (kidney, liver, and spleen) was performed to confirm the biosafety of scaffolds. No abnormality, inflammation, or organ damage was observed in different scaffold-treated groups, proving the acceptable safety and histocompatibility of the scaffolds (Fig. 5b).

### *3.9* *In vivo* bone formation studies

**Fig. 6:**
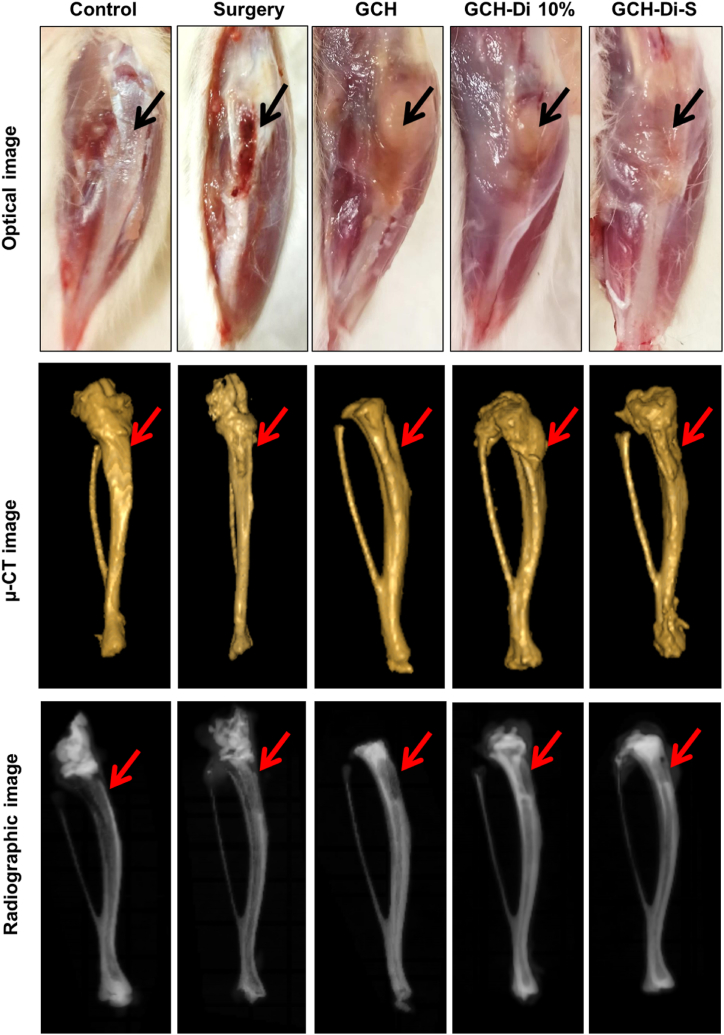
Optical, *μ*-CT, and radiographic images of the control, surgery, GCH, GCH-Di 10%, and GCH-Di-S-treated groups 7 weeks after implantation in the rat tibia defect site. (N = 3).

**Fig. 7:**
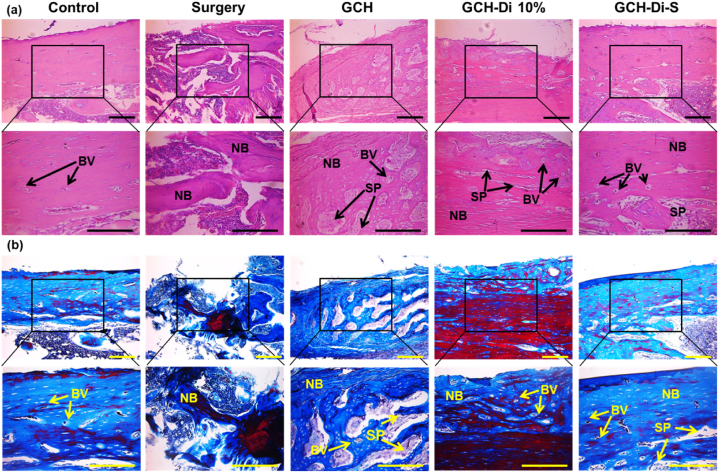
(a) H&E and (b) MT staining of defect sites in control, surgery, GCH, GCH-Di 10%, and GCH-Di-S groups 7 weeks after implantation at × 4 and × 10 magnification. HB=Host Bone, SP=Scaffold Particle, NB=New Bone, and BV=Blood Vessel. Scale bar = 100 μm (N = 3).

To investigate the new bone formation activity of the GCH, GCH-Di 10%, and GCH-Di-S scaffolds, the rat tibia defect model was conducted by creating a hole in the left tibia of rats, followed by scaffold implantation (Fig. S10) and monitoring for 7 weeks. At the end of 3, 5, and 7 weeks, the bones were taken out and evaluated using microscopic, *μ*-CT, and histological analysis (Figs. S11–12 and Fig. 6, Fig. 7). Macroscopic images of the surgery group observed infection in the 3 weeks, as well as all the scaffold-treated groups demonstrated no inflammation or injury during the period study due to the anti-inflammatory activity of CS, Di, and BS in the scaffold.

According to the *μ*-CT images, the groups receiving the scaffolds showed higher new bone formation within 3 weeks after the surgery than surgery group (Fig. S11). After 5 weeks, the GCH scaffold-treated group showed a higher bone repair compared to the surgery-treated group, and the highest amount of new bone formation and density were observed for GCH-Di 10% and GCH-Di-S scaffold-treated groups (Fig. S12). The same results were observed at 7 weeks with, the higher promotion of *de novo* bone formation along the time the holes were almost filled. Overall, the polymeric structure of the GCH scaffold promoted bone formation by providing bridges between the tissues, which increases adhesion and cell proliferation [[85], [86], [87]]. In addition to polymeric structure, the presence of Di enhances calcification, proliferation, and the function of osteoblast, which causes osteogenesis and promotes the healing process [78,88].

The radiographic evaluations confirmed the results of *μ*-CT in all time points of study and demonstrated that the highest new bone formation and density were observed in GCH-Di 10% and GCH-Di-S scaffold-treated groups.

H&E stained defect sites 7 weeks after treatment revealed the lowest bone formation in the surgery group, whereas the GCH-Di 10% and GCH-Di-S groups had the highest new bone formation and blood vessel growth (Fig. 7a). Moreover, the scaffolds of all treated groups were well incorporated into the host tissue, owing to the gradual degradation of the scaffolds proportional of the construction of new bone. The new bone formed showed the structure and morphology similar to the natural bone tissue with calcification of osteocytes in all groups. Furthermore, MT staining demonstrated high collagen deposition in the GCH, GCH-Di 10%, and GCH-Di-S scaffold-treated groups, while loosely formed connective tissue was observed in the surgery group (Fig. 7b). Specifically, newly formed bone almost covered the defect and caused the reconstruction of the trabecular bone mass in the GCH-Di 10% and GCH-Di-S groups, due to the presence of Di and angiogenesis potential of BS. These histological observations confirmed the *in vitro* results that the GCH-Di-S scaffold significantly enhanced osteogenesis with good integrity compared to other groups.

## 4 Conclusions

The open-porous 3D scaffold of GCH-Di-S with interconnected pores was fabricated by freeze-drying. This scaffold exhibited favourable physicochemical attributes, such as porosity of 53–90% (depending on the percentage of the Di in the structure) with interconnected pore sizes of 240 μm, high biocompatibility (hemolytic index 0.11–1.02%), biodegradability, and desirable mechanical strength of 19.3 MPa, providing an appropriate environment for bone regeneration. The addition of Di contributed to enhanced biomineralization (Ca:P ratio 1.84 on day 14), thereby fostering the deposition of hydroxyapatite on the scaffold's surface. Notably, the scaffold demonstrated a potential as a carrier for therapeutic agents, showcasing the ability to locally release BS to harness its anti-inflammatory and angiogenic capabilities. Furthermore, this biosafe scaffold exhibited the capacity to expedite the formation of new bone in a rat tibia defect model, accomplishing this without inducing inflammation at the defect site. These findings underscore the scaffold's potential utility in effective bone repair, attributed to its osteoconductive and osteoinductive properties.

## CRediT authorship contribution statement

**Mina Mohammadi:** Writing – original draft, Methodology, Investigation, Formal analysis, Data curation. **Samin Abbaszadeh:** Validation, Methodology. **Vahideh Nosrati-Siahmazgi:** Validation, Methodology. **Mahsa Akbari:** Methodology. **Saman Rezaei:** Methodology. **Kiyan Musaie:** Methodology. **Mohammad Reza Eskandari:** Resources, Methodology. **Hélder A. Santos:** Writing – review & editing, Resources. **Narges Poursina:** Writing – review & editing, Conceptualization. **Mohammad-Ali Shahbazi:** Writing – review & editing, Supervision, Resources, Investigation, Conceptualization.

## Declaration of competing interest

The authors declare that they have no known competing financial interests or personal relationships that could have appeared to influence the work reported in this paper.

## Abbreviations

BTE: Bone tissue engineering
Gel: gelatin
CS: chitosan
HA: hyaluronic acid
GCH: final scaffold with crosslinked
Di: diatom
BS: β-sitosterol
GCH-Di-S: GCH scaffold with incorporated 10% Di, BS, and crosslinked
EDC: 1-ethyl-(3,3-dimethyl aminopropyl)-carbodiimide
NHS: N-hydroxy succinimide
MES: 2-(N-morpholine) ethane sulfonic acid
SEM: scanning electron microscopy
EDX: energy dispersive X-ray spectra
FT-IR: fourier-transform infrared spectroscopy
XRD: X-ray diffraction spectrometry
TGA: thermal gravimetric analysis
DTG: derivative thermogravimetry
PBS: phosphate buffer saline
RBCs: red blood cells
SBF: simulated body fluid
μ-CT: micro-computed tomography
H&E, RBCs: hematoxylin and eosin
MT: Masson's Trichrome
