# Supplementary material for: Diatom-guided bone healing via a hybrid natural scaffold
Source: Heliyon. 2024 Feb 11;10(4):e25878. doi: 10.1016/j.heliyon.2024.e25878 (PMC10878915; doi:10.1016/j.heliyon.2024.e25878)

**Electronic Supporting Information**

**Diatom-guided bone healing via a hybrid natural scaffold**

Mina Mohammadi^a^, Samin Abbaszadeh^b,c^, Vahideh Nosrati-Siahmazgi^a^, Mahsa Akbari^d^, Saman Rezaei^a^, Kiyan Musaie^e^, Mohammad Reza Eskandari^f^, Hélder A. Santos***^e,g^, Narges Poursina**^a,h^, and Mohammad-Ali Shahbazi*^e^

^a^ Department of Pharmaceutical Biomaterials, School of Pharmacy, Zanjan University of medical Science, 45139-56184 Zanjan, Iran

^b^ Department of Pharmacology, School of Medicine, Zanjan University of medical Science, 45139-56111 Zanjan, Iran

^c^ Department of pharmacology and Toxicology, school of pharmacy, Urmia University of medical sciences, Urmia, Iran

^d^ Department of Pharmaceutical Nanotechnology, School of Pharmacy, Zanjan University of medical Science, 45139-56184 Zanjan, Iran

^e^ Department of Biomaterials and Biomedical Technology, University Medical Center Groningen, University of Groningen, Antonius Deusinglaan 1, 9713 AV Groningen, The Netherlands

^f^ Department of Toxicity, School of Pharmacy, Zanjan University of medical Science, 45139-56184 Zanjan, Iran

^g^ Drug Research Program, Division of Pharmaceutical Chemistry and Technology, Faculty of Pharmacy, University of Helsinki, Helsinki FI-00014, Finland

^h^ Department of Pharmaceutics, School of Pharmacy, Zanjan University of Medical Science, 45139-56184 Zanjan, Iran.

*Correspondence to: [m.a.shahbazi@umcg.nl](mailto:m.a.shahbazi@umcg.nl); [n.poursina@zums.ac.ir](mailto:n.poursina@zums.ac.ir); [h.a.santos@umcg.nl](mailto:h.a.santos@umcg.nl)

**Supplementary Results**


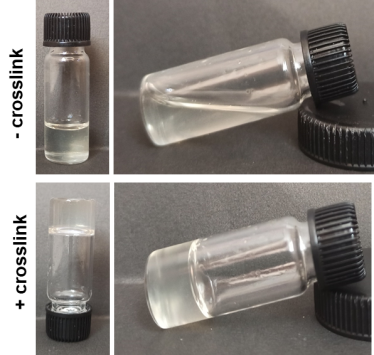


**Figure S1:** Photographs of GCH hydrogel in the presence and absence of EDC/NHS as the crosslinker.


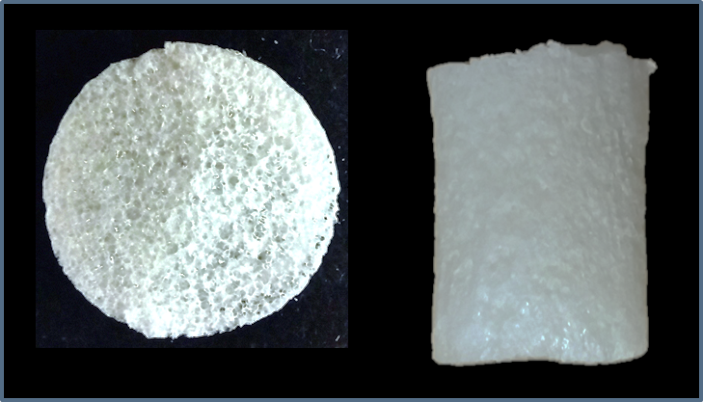


**Figure S2:** Macroscopic images of GCH scaffold in different viewpoints. The GCH represent the final scaffold with the crosslinker.


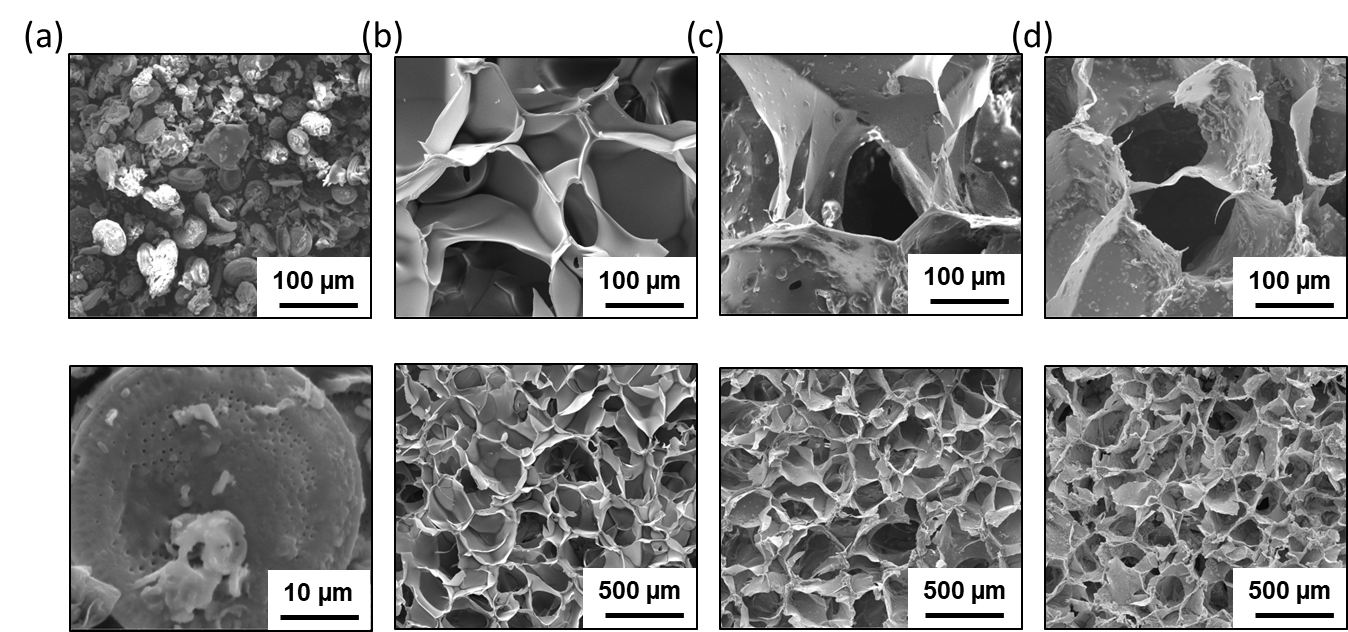


**Figure S3:** SEM images of a) Di, b) GCH, c) GCH-Di 2%, and d) GCH-Di 5% in two different magnifications. The GCH-Di 2% represents the crosslinked GCH scaffold with 2% Di and GCH-Di 5% represent the crosslinked GCH scaffold with 5% Di.


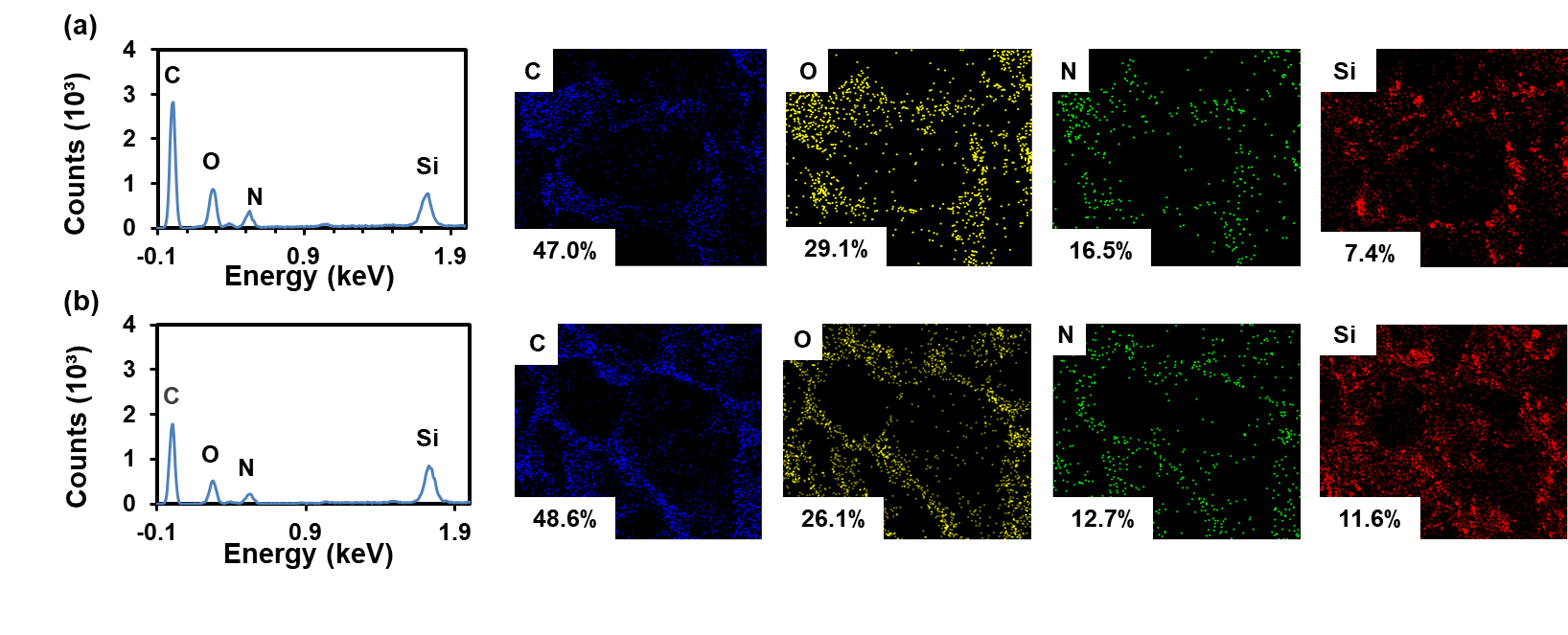


**Figure S4:** EDX analysis and elemental mapping and of a) the GCH-Di 2% and b) the GCH-Di 5% scaffolds.


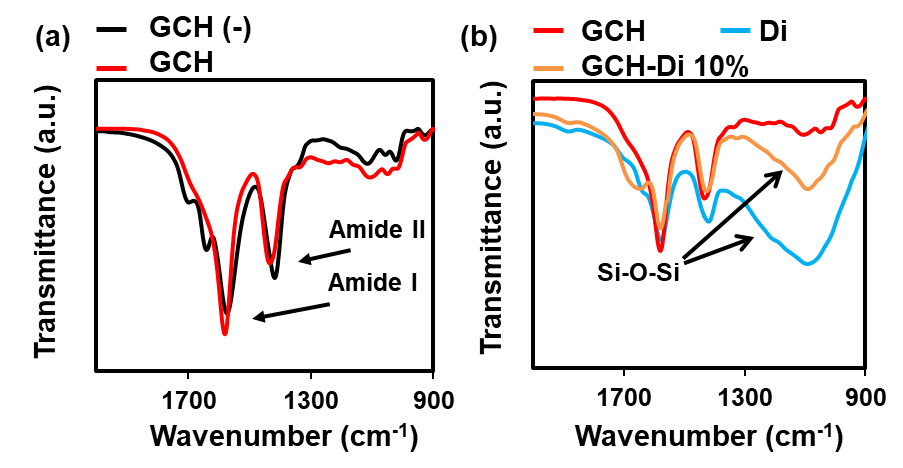


**Figure S5:** FT-IR spectra of a) the GCH (-) and GCH scaffolds. b) FT-IR spectra of the Di, GCH, and GCH-Di 10% scaffolds. The GCH (-) represents the GCH without crosslinker.


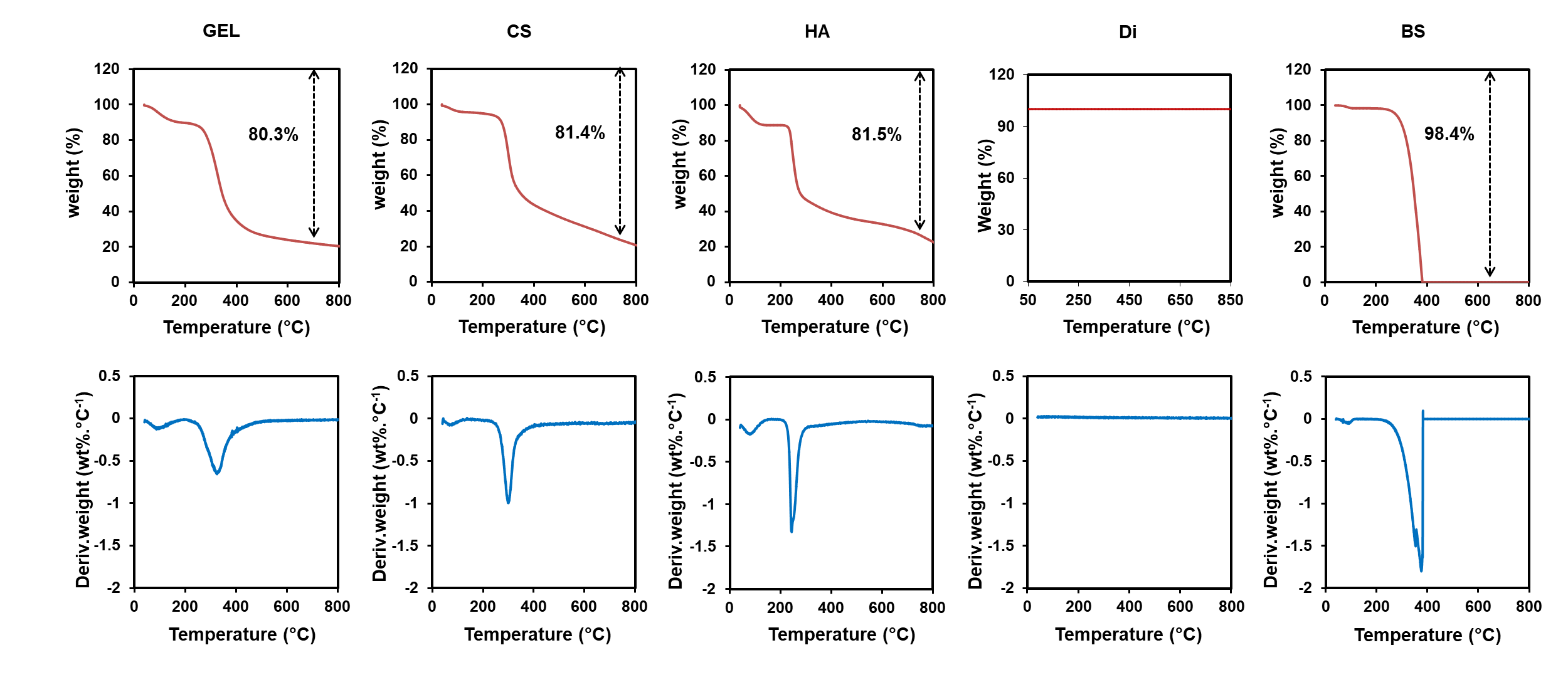


**Figure S6:** TGA (top) and DTG (bottom) results of GEL, CS, HA, Di, and BS.


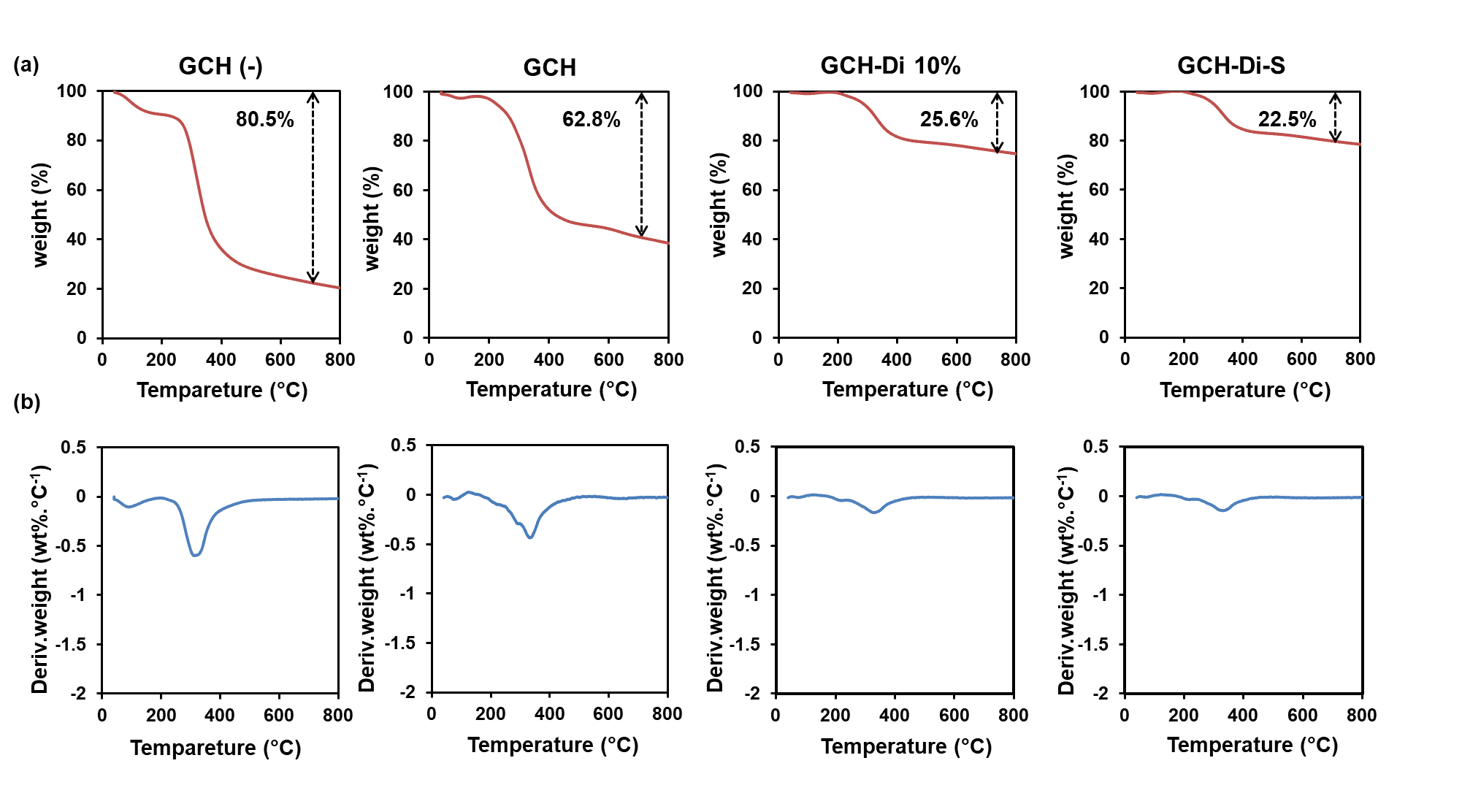


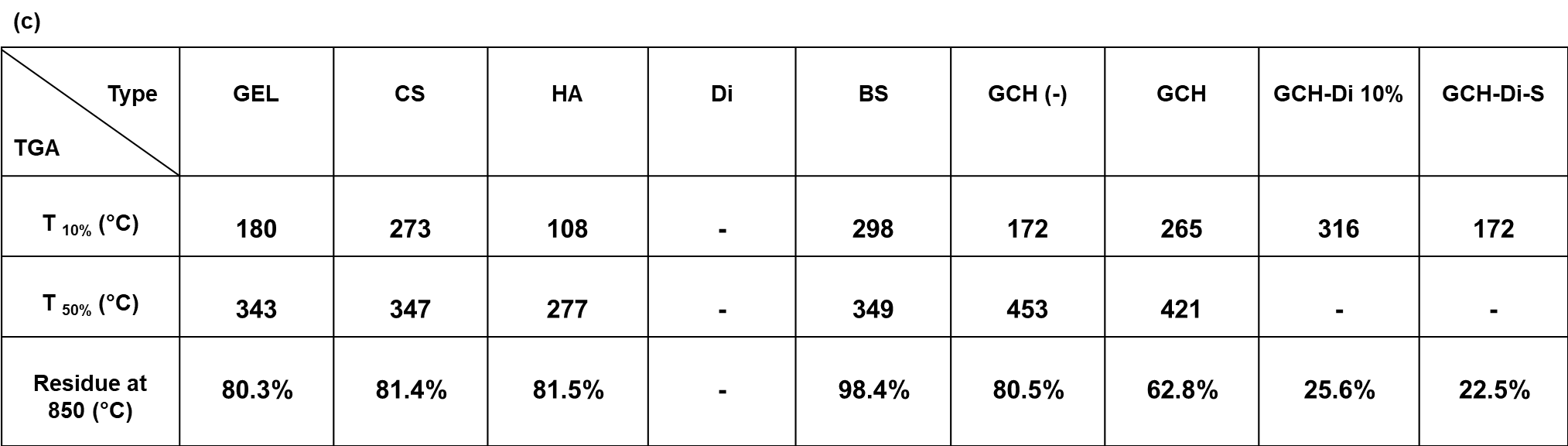


**Figure S7:** a-b) TGA and DTG results of GCH (-), GCH, GCH-Di 10%, and GCH-Di-S. c) T10% (temperature (°C) at 10% weight loss), T50% (temperature (°C) at 50% weight loss), and residual content at 850 °C retrieved from TGA. The GCH-Di-S represents the GCH scaffold with incorporated 10% Di, BS, and the crosslinker.


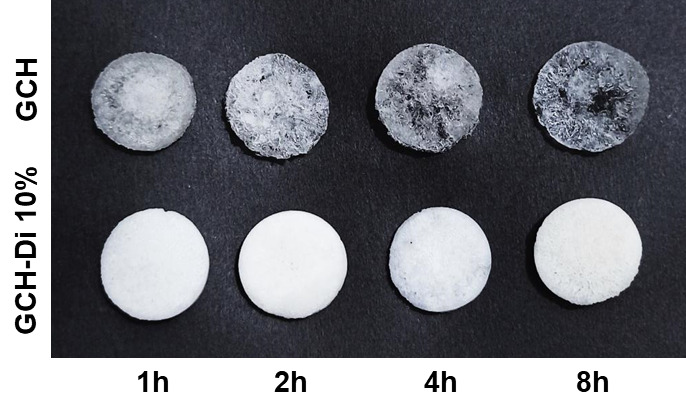


**Figure S8:** Photographs of swelling of GCH and GCH-Di 10% scaffolds at different times.


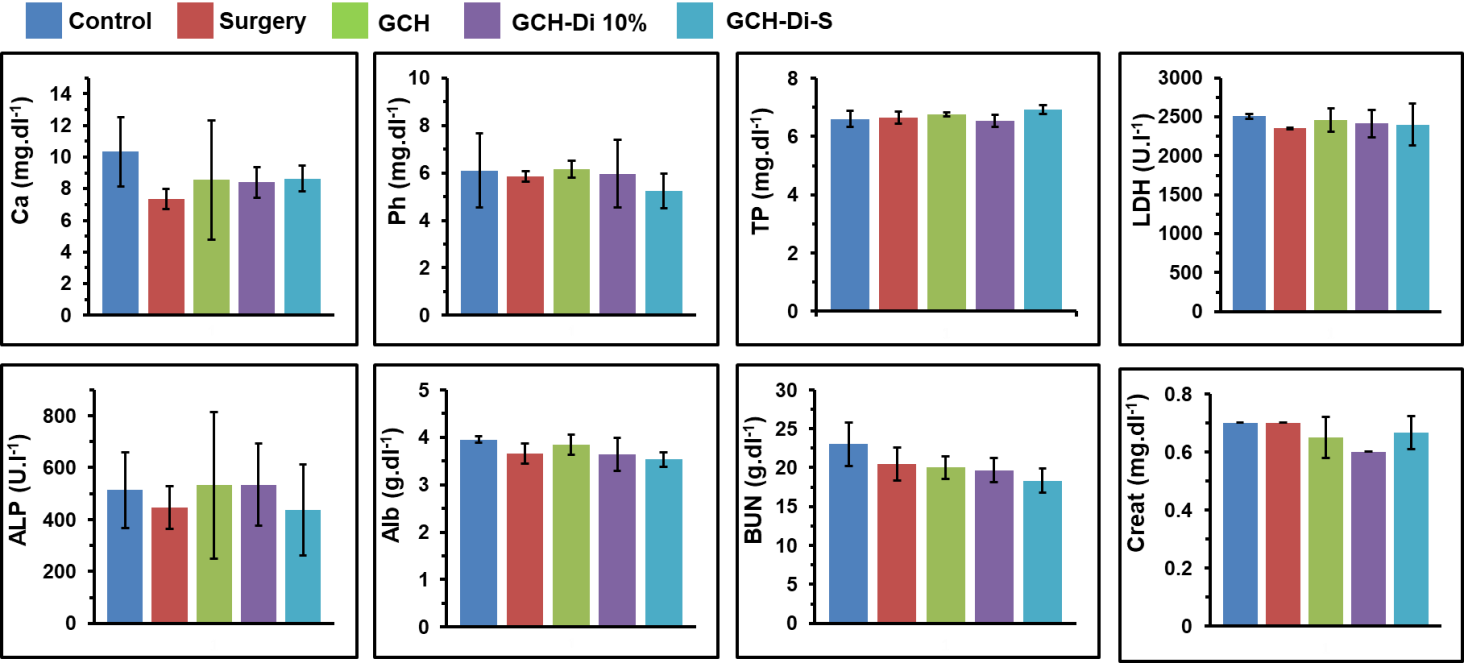


**Figure S9:** *In vivo* toxicity analysis. Biochemical parameters after 5 weeks placing the implants (GCH, GCH-Di 10% and GCH-Di-S) in the defect site of the tibia bone in comparison to control healthy animals and surgery group (defect creation without implantation). Data are represented as mean±SD (N=3). Statistical analysis calculated by one way ANOVA with the level of significance set at * p<0.05, ** p<0.01, *** p<0.001.


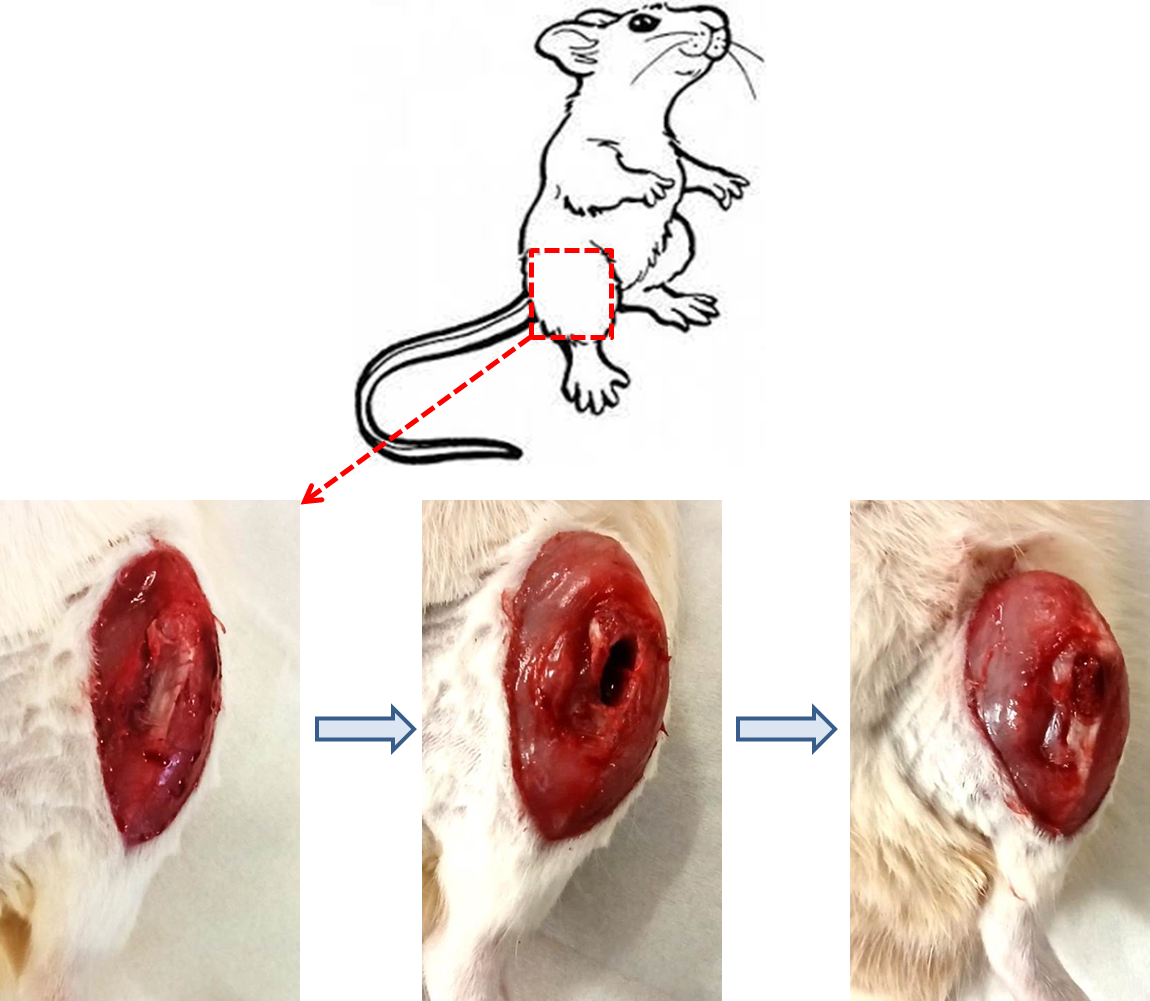


**Figure S10:**  Images of the surgical process and scaffold implantation in the rat tibia.


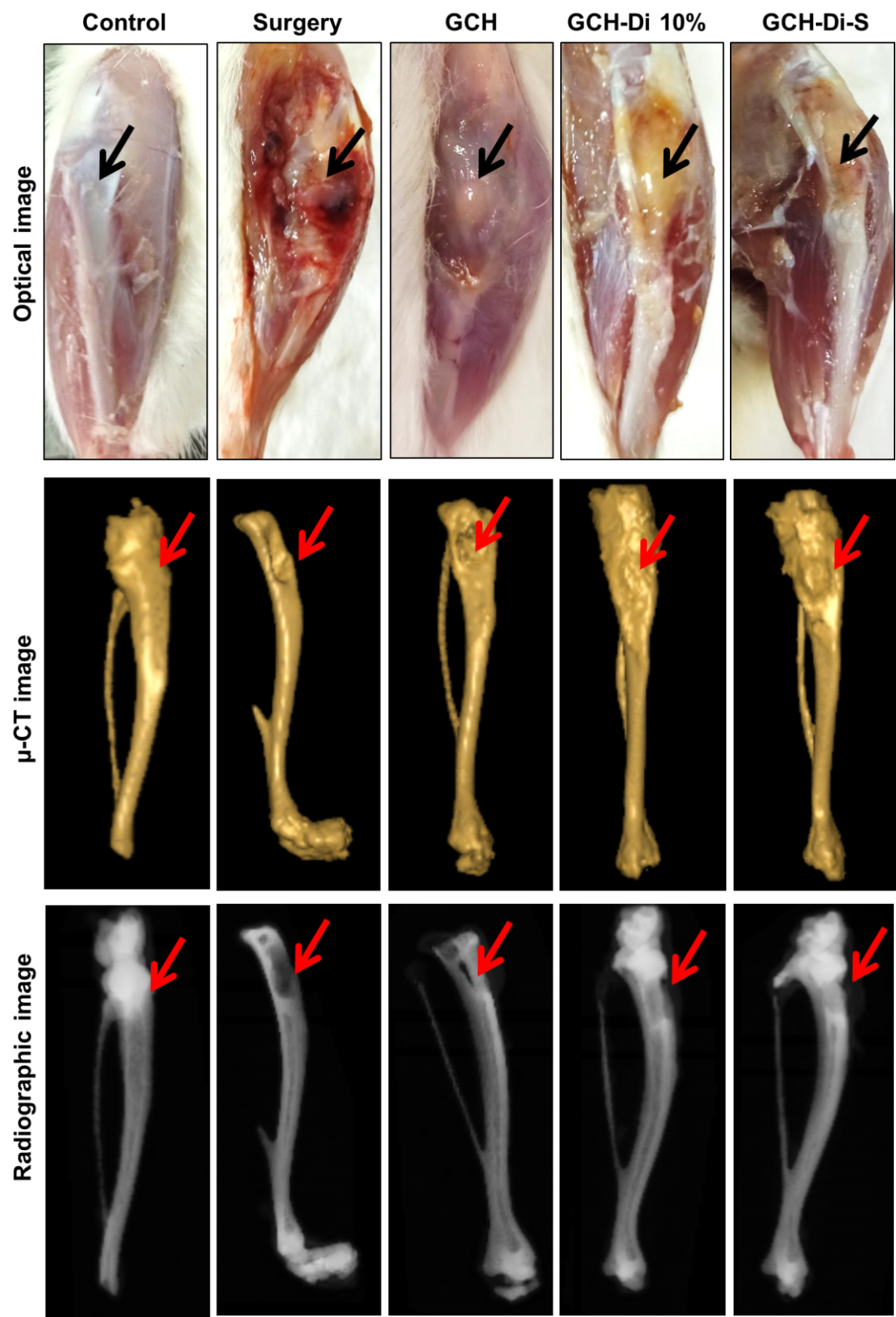


**Figure S11:** Optical, µ-CT, and radiographic images of the control, surgery, GCH, GCH-Di 10%, and GCH-Di-S-treated groups three weeks after implantation in the defect site of tibia in rats. (N=3).


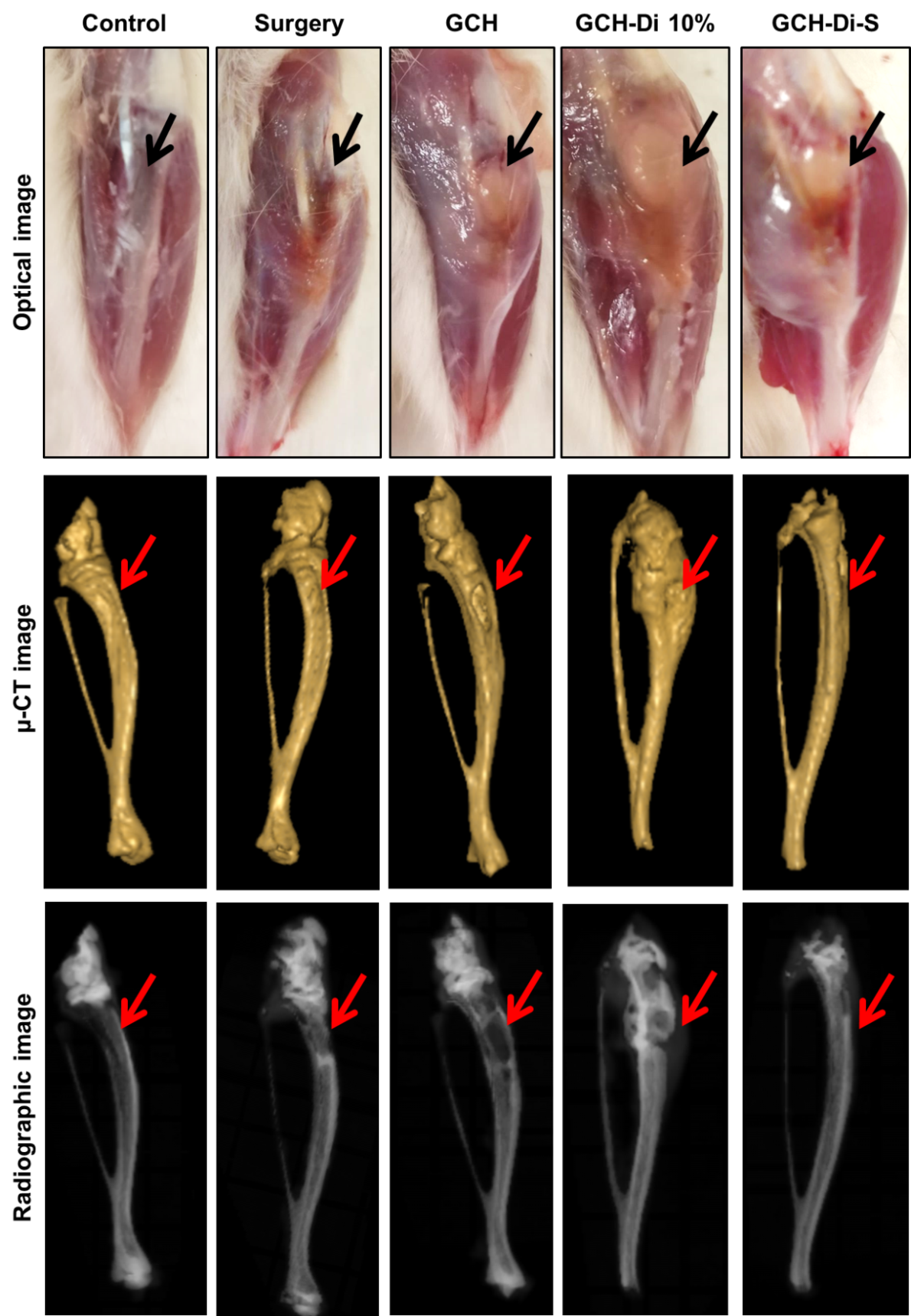


**Figure S12:** Optical, µ-CT, and radiograpic images of the control, surgery, GCH, GCH-Di 10%, and GCH-Di-S-treated groups five weeks after implantation in the defect site of tibia in rats. (N=3).

**Table S1:** EDX results of the GCH and GCH-Di with 2, 5, and 10% of Di in the scaffolds. The GCH-Di 10% represents the crosslinked GCH scaffold with 10% Di .

| **Scaffold**  **Elemen**t | **GCH** | **GCH-Di 2%** | **GCH-Di 5%** | **GCH-Di 10%** |
| --- | --- | --- | --- | --- |
| **C** | 47.8 | 47.0 | 48.6 | 40.0 |
| **O** | 31.6 | 29.1 | 26.1 | 31.0 |
| **N** | 20.6 | 16.5 | 12.7 | 10.5 |
| **Si** | - | 7.4 | 11.6 | 18.5 |

**Table S2:** FTIR peaks assignment.

| **Wave number (cm^-1^)** | **Assignment** |
| --- | --- |
| 1583, 1440 | C=O stretching vibration of carboxylate |
| 1571 | N-H stretching of amine |
| 1000-1200 | C-O stretching vibration of alcohol I & II |
| 1600 | C-O stretching vibration carboxylate |
| 1573, 1581 | C-N amide I |
| 1417, 1435 | C-N amide II |
| 1090 | Si-O-Si asymmetric stretching vibration |
| 1640 | O-H bending vibration |
| 1641 | C=C Stretching vibration |

**Table S3:** XRD peaks of the pure materials (GEL, CS, HA, Di, and BS), GCH (-), GCH, GCH-Di 10%, and GCH-Di-S scaffolds.

| **Sample** | **2θ(°) amorph** | **2θ(°) semi** | **2θ(°) semi** | **2θ(°) crystal** | **2θ(°) crystal** |
| --- | --- | --- | --- | --- | --- |
| **GEL** | 20.1 | - | - | - | - |
| **CS** | - | 10.5 | 20.2 | - | - |
| **HA** | 20 | - | - | - | - |
| **Di** | - | - | - | 21.8 | 36.1 |
| **BS** | - | - | - | 15 | 18.3 |
| **GCH(-)** | 20.1 | - | - | - | - |
| **GCH** | 20.1 | - | - | - | - |
| **GCH-Di 10%** | - | - | - | 21.8 | 36.1 |
| **GCH-Di-S** | - | - | - | 21.8 | 36.1 |

**Table S4:** EDX results of biomineralization on the GCH-Di-S scaffold.


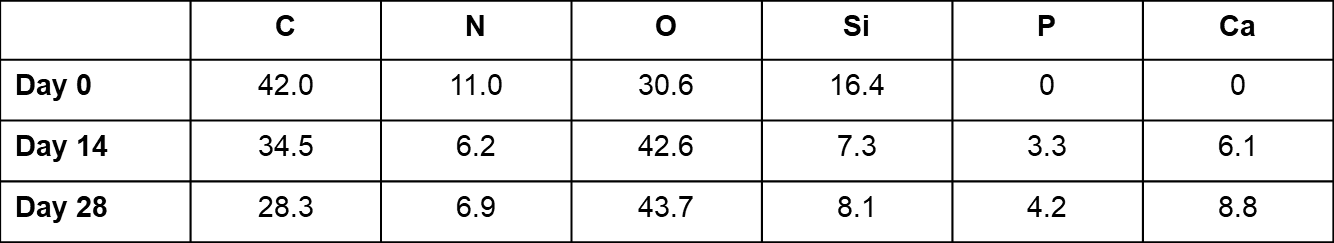

Supplement: Multimedia component 1 [file mmc1.docx]
